# Role of Thyrotropin-Releasing Hormone Stimulation Test and Autoantibody in 952 Subjects with Subclinical Hypothyroidism

**DOI:** 10.1210/jendso/bvae212

**Published:** 2024-12-12

**Authors:** Myung Hi Yoo, Hye Jeong Kim, Suyeon Park, Sang Joon Park, Hyeong Kyu Park, Dong Won Byun, Kyoil Suh

**Affiliations:** Division of Endocrinology and Metabolism, Department of Internal Medicine, Soonchunhyang University Hospital, Soonchunhyang University College of Medicine, Seoul 04401, Korea; Elim Thyroid Clinic, Hanshin Medipia, Seoul 06520, Korea; Division of Endocrinology and Metabolism, Department of Internal Medicine, Soonchunhyang University Hospital, Soonchunhyang University College of Medicine, Seoul 04401, Korea; Department of Biostatistics, Academic Research Office, Soonchunhyang University Seoul Hospital, Seoul 04401, Korea; International Development and Cooperation, Graduate School of Multidisciplinary Studies Toward Future, Soonchunhyang University, Asan 31538, Korea; Department of Applied Statistics, Chung-Ang University, Seoul 06974, Korea; Division of Endocrinology and Metabolism, Department of Internal Medicine, Soonchunhyang University Hospital, Soonchunhyang University College of Medicine, Seoul 04401, Korea; Division of Endocrinology and Metabolism, Department of Internal Medicine, Soonchunhyang University Hospital, Soonchunhyang University College of Medicine, Seoul 04401, Korea; Division of Endocrinology and Metabolism, Department of Internal Medicine, Soonchunhyang University Hospital, Soonchunhyang University College of Medicine, Seoul 04401, Korea; Division of Endocrinology and Metabolism, Department of Internal Medicine, Soonchunhyang University Hospital, Soonchunhyang University College of Medicine, Seoul 04401, Korea

**Keywords:** subclinical hypothyroidism, hypothyroidism, thyrotropin-releasing hormone stimulation test, TPOAb, TgAb

## Abstract

**Context:**

Subclinical hypothyroidism (SCH) is characterized by elevated thyroid-stimulating hormone (TSH) levels and normal free thyroxine (fT4) levels. In upper normal TSH levels, thyrotropin-releasing hormone (TRH) stimulation test proved to be useful in identifying an exaggerated TSH response.

**Objective:**

We aimed to evaluate the incidence and predictive ability of basal TSH, anti-thyroid peroxidase antibodies (TPOAb), and anti-thyroglobulin antibodies (TgAb) for exaggerated TRH stimulation test in SCH.

**Methods:**

A total of 952 subjects with SCH (TSH 4.01-10.00 mIU/L) found during health checkups were evaluated for TSH response to TRH stimulation testing and autoantibodies. Exaggerated TSH response was defined as ΔTSH (peak serum TSH level after TRH injection minus basal TSH level) of > 25.00 mIU/L.

**Results:**

The prevalence of exaggerated TSH responses in SCH was 66% (n = 633). The proportion of exaggerated TSH response tended to increase as basal TSH levels increased (*P* for trend <.001). Also, the proportion of positive TPOAb or TgAb tended to increase as basal TSH levels increased (*P* for trend <.05). Analysis of predictive ability of basal TSH, positive TPOAb, or TgAb for exaggerated TRH stimulation test revealed that positive TPOAb or TgAb showed high specificity (> 90%) for positive TRH stimulation test but low sensitivity. Basal TSH showed low sensitivity and specificity.

**Conclusion:**

Two-thirds of SCH showed exaggerated TRH stimulation test. Positive TPOAb or TgAb showed high specificity (> 90%) for positive TRH stimulation test but basal serum TSH levels showed low predictability. The TRH stimulation test may be a valuable guide to identify SCH patients with hypothyroid state.

Subclinical hypothyroidism (SCH) is characterized by a serum thyroid-stimulating hormone (TSH) above the upper reference limit in combination with a normal free thyroxine (fT4). SCH is a highly prevalent endocrine disorder, with population prevalence ranging from 3% to 10% depending on factors such as sex, age, and the specific population under study [1-5]. SCH was associated with a significant risk for coronary heart disease, cardiovascular mortality [6-9] and infertility [10, 11]. However, there is limited evidence that treatment of SCH improves clinical outcomes [12-17], resulting in controversy over benefit of treatment of SCH. In the current guidelines for treatment of SCH, thyroid hormone treatment is not routinely recommended [18-20]. But active case finding to avoid the possible risk of SCH is usually encouraged [21, 22]. According to the joint statement of the Consensus Development Conference sponsored by the American Association of Clinical Endocrinologists, the American Thyroid Association, and the Endocrine Society, which was held in September 2002 [21], Gharib et al suggested evidence for treating patients with TSH levels above 10.00 mIU/L is compelling, but also most patients with serum TSH levels of 4.50 to 10.00 mIU/L should be considered for treatment because the lack of definite evidence for a benefit does not equate to evidence for lack of benefit.

In SCH, as long as the serum TSH level is elevated, the thyroid hormone levels are not truly normal and elevated TSH in a patient means that the circulating thyroid hormone concentrations are insufficient [22] for the normal negative feedback to the pituitary gland. Also, an increase of serum TSH requires repeated measurements because several factors such as circadian fluctuations, increasing age, and delay in the nocturnal peak of serum TSH had been shown to lead to transient abnormalities of serum TSH [23, 24]. The thyrotropin-releasing hormone (TRH) stimulation test is known to be valuable to identify an exaggerated TSH response in the upper normal TSH levels [25-28].

The role of autoantibodies and higher basal TSH level to increase the incidence of future hypothyroidism in SCH was reported in several cohort studies [2, 5, 29]. And the presence of positive autoantibodies was reported to increase the development of hypothyroidism [2, 29]. But whether basal TSH and anti-thyroid peroxidase antibodies (TPOAb) or anti-thyroglobulin antibodies (TgAb) would be valuable to predict present exaggerated TRH stimulation test has not been addressed. We aimed to evaluate the incidence of an exaggerated TSH response to the TRH stimulation test in SCH and the incidence of thyroid autoantibody in SCH. And we tried to evaluate the predictive ability of basal TSH, TPOAb, and TgAb for exaggerated TRH stimulation test in SCH.

## Methods

### Subjects

We retrospectively reviewed the medical records of 1027 consecutive patients aged ≥ 19 years who visited the thyroid clinic of Soonchunhyang University Hospital (between April 2003 and March 2021, n = 547) and the Elim Thyroid Clinic (between May 2019 and October 2022, n = 580) for the evaluation of SCH during health checkups and who consented to undergo the TRH stimulation test. Of these, 75 subjects with initial TSH levels of > 10.00 mIU/L were excluded, and thus, 952 subjects were available for analysis of TSH response in TRH stimulation test. None of subjects were pregnant or taking any medication known to influence on thyroid function, such as levothyroxine, anti-thyroid drugs, iodine, or lithium. Among 952 participants, evaluation of both TPOAb and TgAb was available in 850 subjects. Data analysis was performed in 952 participants regarding basal TSH and TRH stimulation test. Analysis including thyroid autoantibodies was done in 850 subjects with available autoantibody data.

Informed consent requirement for this study was waived by the institutional review board because researchers only accessed the database for analysis purposes, and personal identifying information was not accessed. The Institutional Review Board of Soonchunhyang University Hospital approved the study (IRB File No. 2021-11-010).

### Laboratory Assay

Serum TSH were measured using the immunoradiometric assay using a TSH-CTK-3 RIA kit (DiaSorin SpA, Saluggia, Italy, cat# 209-100, RRID: AB_3662704) with a laboratory reference range of 0.30 to 4.00 mIU/L and with interassay and intraassay variances of < 10% and 5%, respectively. A TRH stimulation test was performed between 9:00 Am and 12:00 Pm at the next visit after the initial TSH level was measured. Serum TSH levels were measured immediately prior to the intravenous TRH administration and the TSH levels were assessed again 30, 45, 60, 90 and 120 minutes after intravenous injection of 400 μg TRH (Aventis, Frankfurt, Germany).

Serum fT4 levels were assessed using a radioimmunoassay with an FT4 RIA kit (Immunotech, Prague, Czech Republic, cat# IM1363 RRID: AB_2895185), with a laboratory reference range of 0.89 to 1.78 ng/dL.

Serum TPOAb levels were measured using a RIA kit (BRAHMS, Hennigsdorf, Germany, cat# BR-901, RRID: AB_3662644) with a laboratory reference range of 0.00 to 60.00 IU/mL. Serum TgAb levels were measured using a radioimmunoassay (RSR Ltd, Cardiff, UK, cat# KTG1347, RRID:AB_3662645) with a laboratory reference range of 0.00 to 60.00 IU/mL.

### Definitions

SCH was defined as TSH levels 4.01 to 10.00 mIU/L and normal fT4 levels, referring to the definitions of previous studies [1, 20]. An exaggerated TSH response was defined as a ΔTSH (defined as a peak serum TSH level after TRH injection minus a basal TSH level) of > 25.00 mIU/L. Thyroid autoimmunity was defined as the presence of TPOAb (≥ 60.00 IU/mL) or TgAb (≥ 60.00 IU/mL).

### Statistical Analysis

Continuous variables were reported as medians with interquartile ranges, and categorical variables were presented as N and percentages (%). The demographic and biochemical characteristics of the subjects with respect to the results of TRH stimulation test were compared using the Mann-Whitney U-test for continuous variables and the χ2 test for categorical variables. Correlations between the basal TSH and ΔTSH in the TRH stimulation test were made using Spearman correlation analysis. The median ΔTSH among the basal TSH groups were compared using the Kruskal-Wallis test. To solve the multiple testing problem, the Bonferroni correction method was performed. The proportions of exaggerated TSH response in the TRH stimulation test with respect to the basal TSH groups were compared using the trend test. Lastly, receiver operating characteristic (ROC) curve analysis was used to confirm the predictive ability of baseline TSH and TPOAb or TgAb for the exaggerated TRH stimulation test.

All statistical analyses were performed using SPSS Statistics version 26.0 (IBM Corp., Chicago, IL, USA) and R ver3.6.3 (https://www.r-project.org/). Item analysis with two-sided *P* value <.05 were considered statistically significant

## Results

### Baseline Characteristics of Subclinical Hypothyroid Subjects With Respect to the Results of TRH Stimulation Test

The baseline clinical and biochemical characteristics of the 952 subjects are summarized in Table 1. The overall prevalence of exaggerated TSH response in the TRH stimulation test was 66% (n = 633), with a higher prevalence in women. Compared to subjects with normal response in TRH stimulation test, exaggerated TSH response were more frequent in women, and subjects with higher basal TSH levels and positive TPOAb or TgAb (Table 1).

**Table 1.** Baseline characteristics of subclinical hypothyroid subjects with respect to the results of TRH stimulation test and autoantibody

	Overall (N = 850)	TRH stimulation test *^a^*		
		Normal response (n = 293)	Exaggerated response (n = 557)	*P* value
Age	46 [36, 55]	46 [35, 56]	47 [3 755]	.665
Sex				
Male	348 (40.94%)	179 (61.09%)	169 (30.34%)	<.001
Female	502 (59.06%)	114 (38.91%)	388 (69.66%)	
TSH (ulU/mL)	5.53[4.73,6.63]	5.24 [4.62,6.21]	5.66 [4.82, 6.88]	<.001
TPOAb				
Negative	714 (84%)	265 (90.44%)	499 (80.61%)	<.001
Positive	136 (16%)	28 (9.56%)	108 (109.39%)	
TgAb				
Negative	709 (83.41%)	266 (90.78%)	443 (79.53%)	<.001
Positive	141 (16.59%)	27 (9.22%)	114 (20.47%)	
Thyroid autoimmunity				
Negative	633 (74.47%)	250 (85.32%)	383 (68.76%)	<.001
Positive	217 (25.53%)	43 (14.68%)	174 (31.24%)	

Data are presented as numbers (percentage) or medians [25th, 75th percentiles] as appropriate for the variable. Demographic and biochemical characteristics of the study population with the results of TRH stimulation test were compared using χ^2^ test for categorical variables and the Mann-Whitney U-test for continuous variables.

Abbreviations: fT4, free thyroxine; TgAb, anti-thyroglobulin antibody; TPOAb, anti-thyroid peroxidase antibody; TRH, thyrotropin-releasing hormone; TSH, thyroid-stimulating hormone.

^*a*^ΔTSH (defined as a peak serum TSH level after TRH injection minus a basal TSH level) > 25.00 mIU/L was regarded as exaggerated TRH stimulation test.

### The Distribution of Subjects in SCH Based on Basal TSH Levels

**Figure 1.**
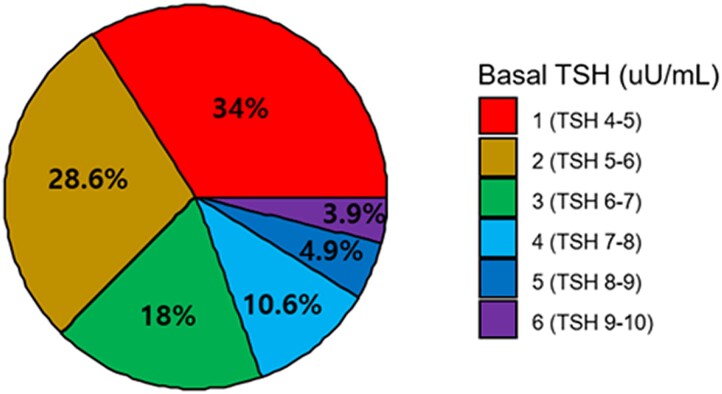
The distribution of the subjects with subclinical hypothyroidism ln 6 groups divided according to the basal TSH levels.

When the subjects were divided according to the basal TSH levels (Group1 with TSH 4.01-5.00, Group2 with TSH 5.01-6.00, Group3 with TSH 6.01-7.00, Group4 with TSH 7.01-8.00, Group5 with TSH 8.01-9.00, and Group6 with TSH 9.01-10.00 mIU/L). Group1 with basal TSH 4.01-5.00 mIU/L comprised 34% of SCH, Group2 with basal TSH 5.01-6.00 mIU/L comprised 28.6% of SCH, and the rest 37.4% of SCH were composed by Groups 3, 4, 5, and 6 (Fig. 1).

### Correlation Between Basal TSH and ΔTSH in the TRH Stimulation Test

**Figure 2.**
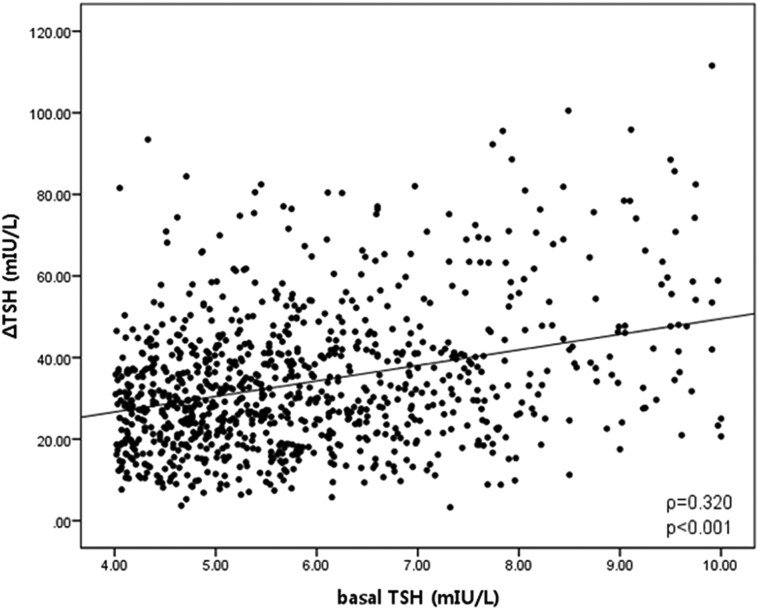
Correlation between basal TSH and ΔTSH in the TRH stimulation test. ΔTSH was defined as a peak serum TSH level after TRH injection minus a basal TSH level.

**Figure 3.**
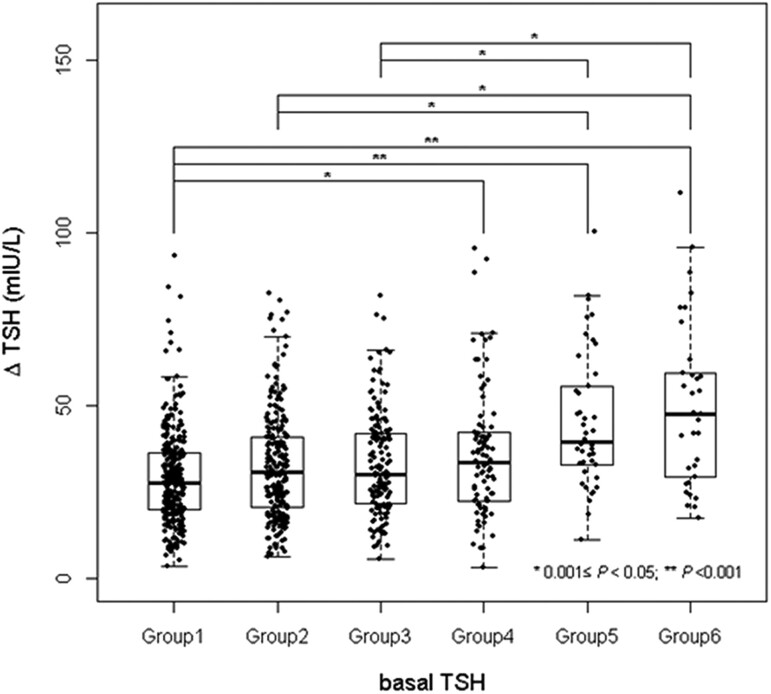
Box-and-whisker plots of ΔTSH in 6 groups divided by the basal TSH levels. ΔTSH was defined as a peak serum TSH level after TRH injection minus a basal TSH level.

As a result of evaluating the correlation between basal TSH and ΔTSH in the TRH stimulation test, ΔTSH showed a weak positive correlation with the basal TSH (*r* = 0.320, *P* < .001, Fig. 2). When the subjects were divided into the 6 Groups according to the basal TSH levels, it was found that the ΔTSH increased as the basal TSH levels increased (*P* for trend <.001) (Table 2, Fig. 3) and more than 80% of subjects with basal TSH levels > 8.00 mIU/L showed an exaggerated TSH response in the TRH stimulation test.

**Table 2.** TRH stimulation test result and positive autoantibodies in 6 groups according to the basal TSH levels

	Total (N = 850)	Group1 (N = 289)	Group2 (N = 243)	Group3 (N = 153)	Group4 (N = 90)	Group5 (N = 42)	Group6 (N = 33)	*P* value
TRH test								
Negative	293 (34.5%)	119 (41.2%)	86 (35.4%)	50 (32.7%)	28 (31.1%)	5 (11.9%)	5 (15.2%)	<.001
Positive	557 (65.5%)	170 (58.8%)	157 (64.6%)	103 (67.3%)	62 (68.9%)	37 (88.1%)	28 (84.8%)	
TPOAb								
Negative	714 (84%)	247 (85.5%)	210 (86.4%)	130 (8.05%)	71 (78.9%)	37 (88.1%)	19 (57.6%)	.004
Positive	136 (16%)	42 (14.5%)	33 (13.6%)	23 (15.0%)	19 (21.1%)	5 (11.9%)	14 (42.4%)	
TgAb								
Negative	709 (83.4%)	246 (85.1%)	205 (84.4%)	131 (85.6%)	68 (75.6%)	38 (90.5%)	21 (63.6%)	.027
Positive	141 (16.6%)	43 (14.9%)	38 (15.6%)	22 (14.4%)	22 (24.4%)	4 (9.5%)	12 (36.4%)	
AutoAb								
Negative	633 (74.5%)	221 (76.5%)	189 (77.1%)	118 (77.1%)	56 (62.2%)	34 (81.0%)	15 (455%)	.002
Positive	217 (25.5%)	68 (23.5%)	54 (22.2%)	35 (22.9%)	34 (37.8%)	8 (19.0%)	18 (54.5%)	

TRH stimulation test results and positive autoantibodies in 6 groups showed statistically significant increasing tendency of the positive test results with increasing basal TSH for Trend test.

Abbreviations: TgAb, anti-thyroglobulin antibody; TPOAb, anti-thyroid peroxidase antibody; TRH, thyrotropin-releasing hormone.

### Correlation Between Thyroid Autoimmunity and Basal TSH

**Figure 4.**
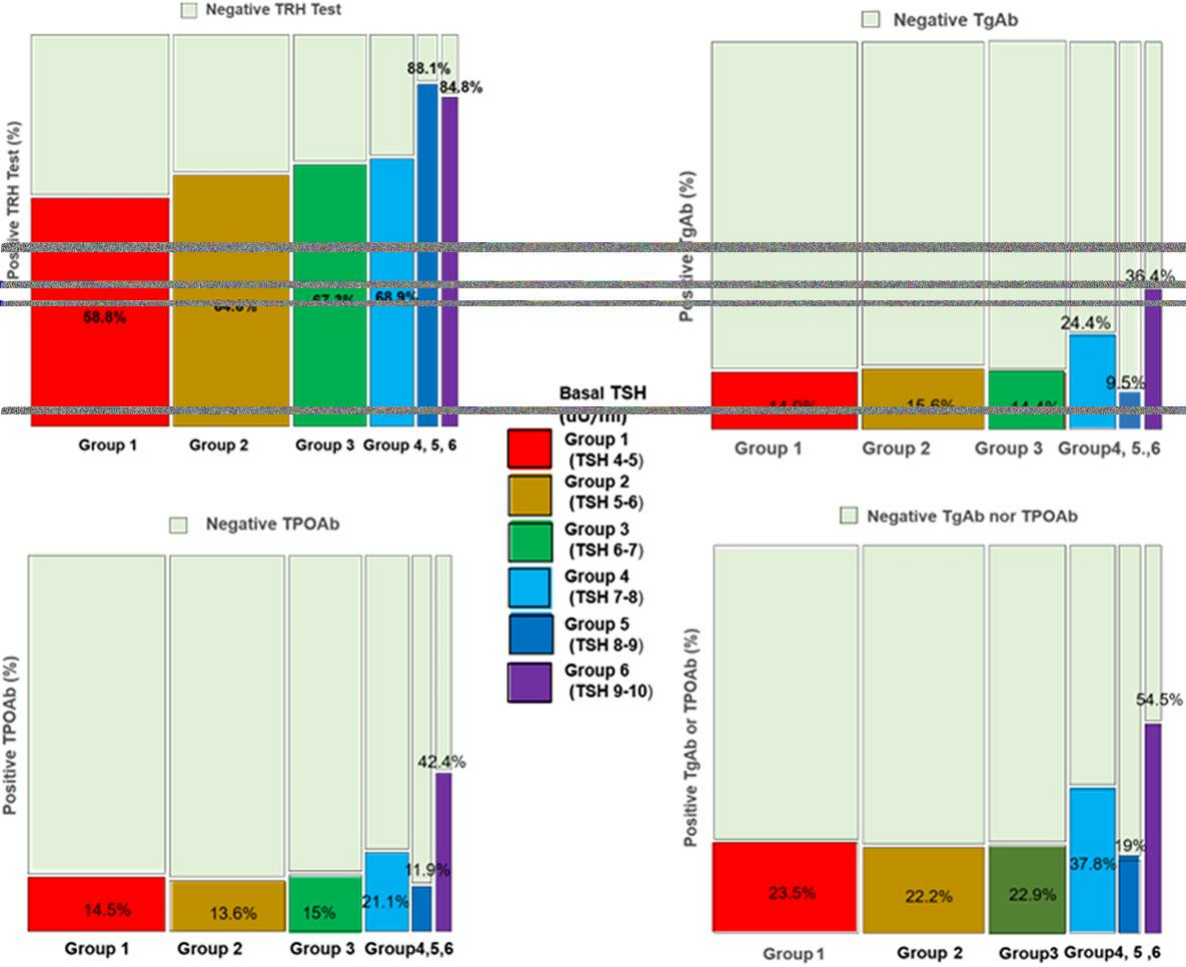
TRH stimulation test result and positive autoantibodies in 6 groups divided by the basal TSH showed statistically significant increasing tendency of the positive results according to the increasing basal TSH levels by Chi-squared test for trend in proportions.

The proportion of positive TPOAb or TgAb tended to increase as basal TSH levels increased (*P* for trend <.05) (Table 2, Fig. 4) and 54.5% of Group6 patients showed positive TPOAb or TgAb.

### Predictive Ability of Basal TSH and TPOAb or TgAb for Exaggerated TRH Stimulation Test

**Figure 5.**
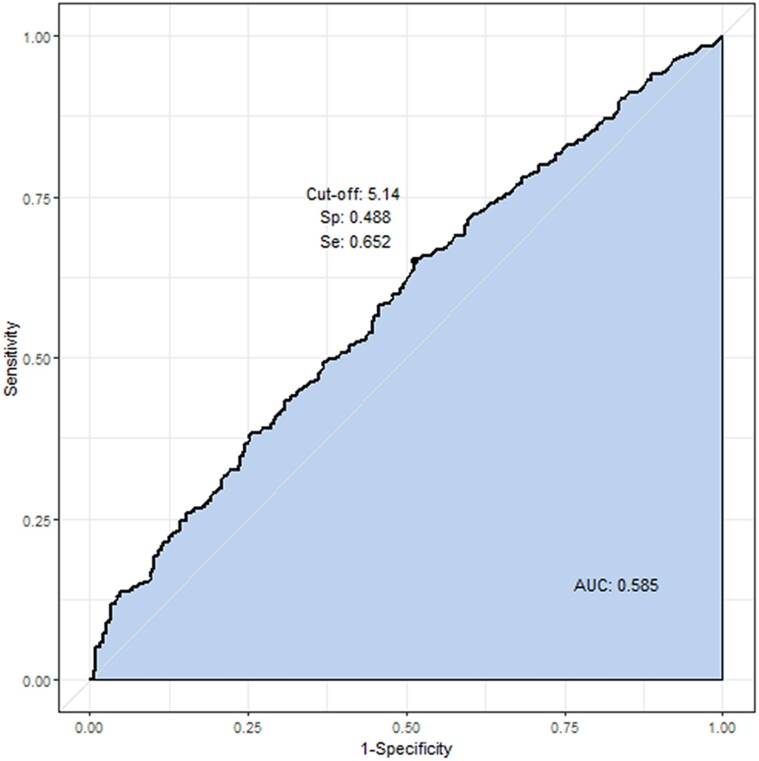
Receiver operating characteristic (ROC) curve analysis of basal TSH to predict positive TRH stimulation test.

**Figure 6.**
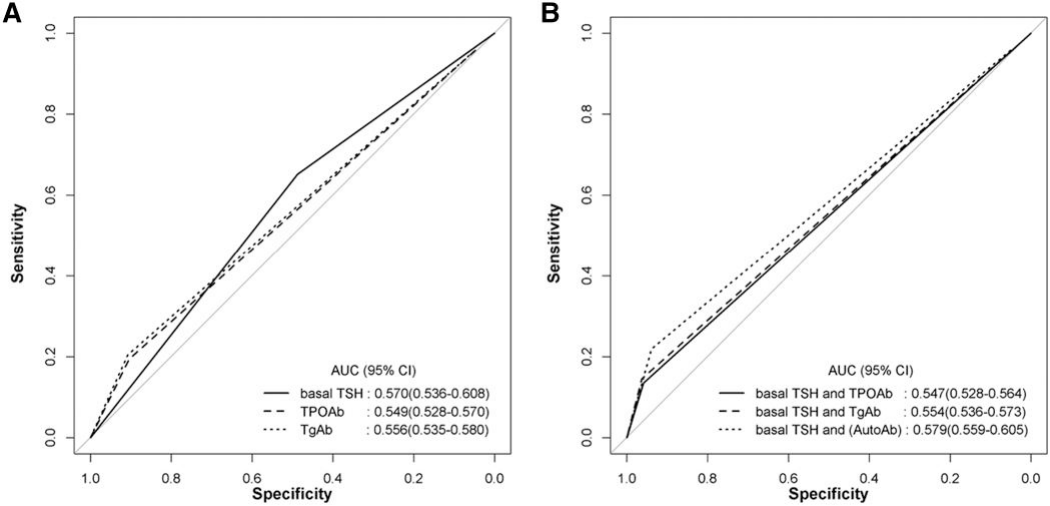
Receiver operating characteristic (ROC) curve analysis of basal TSH, TgAb, and TPOAb to predict positive TRH stimulation test (A). ROC curve analysis of basal TSH with positive TgAb and/or positive TPOAb to predict positive TRH stimulation test (B).

ROC curve analysis of basal TSH to predict positive TRH stimulation test showed a cutoff level of basal TSH of 5.14 mIU/L had 65.2% sensitivity and 48.8% specificity (Fig. 5). ROC curve analysis of positive TPOAb or TgAb to predict positive TRH stimulation test showed high specificity (90.4% and 90.8% respectively (Table 3, Fig. 6) but low sensitivity (19.4% and 20.5% respectively). ROC curve analysis of basal TSH with positive TgAb and/or positive TPOAb to predict positive TRH stimulation test showed similar results as TPOAb or TgAb alone, implying no further gain of predictability by adding the data of basal TSH (Table 3, Fig. 6).

**Table 3.** Predictive ability of basal TSH level and autoantibodies for positive TRH stimulation test

	Sensitivity	Specificity	Accuracy	PPV	NPV
TSH	0.652 (0.612-0.691)	0.488 (0.430-0.547)	0.595 (0.561-0.629)	0.708 (0.666-0.747)	0.424 (0.371-0.479)
TPOAb	0.194 (0.162-0.229)	0.904 (0.865-0.936)	0.439 (0.405-0.473)	0.794 (0.716-0.859)	0.371 (0.336-0.408)
TgAb	0.205 (0.172-0.241)	0.908 (0.869-0.938)	0.447 (0.413-0.481)	0.809 (0.734-0.870)	0.375 (0.339-0.412)
TSH and TgAb	0.145 (0.117-0.178)	0.963 (0.934-0.981)	0.427 (0.394-0.461)	0.880 (0.796-0.939)	0.372 (0.338-0.408)
TSH and TPOAb	0.135 (0.107-0.166)	0.959 (0.930-0.979)	0.419 (0.385-0.453)	0.862 (0.772-0.927)	0.368 (0.334-0.404)
TSH and either TgAb or TPOAb	0.219 (0.185-0.256)	0.939 (0.905-0.963)	0.467 (0.433-0.501)	0.871 (0.804-0.922)	0.387 (0.351-0.424)

Numbers in the parentheses represent 95% CI.

Abbreviations: NPV, negative predictive value; PPV, positive predictive value; TgAb, anti-thyroglobulin antibody; TPOAb, anti-thyroid peroxidase antibody; TRH, thyrotropin-releasing hormone; TSH, thyroid-stimulating hormone.

## Discussion

In our study, approximately two-thirds (66%, 633 out of 952) of SCH patients with TSH levels between 4.01 and 10.00 mIU/L demonstrated an exaggerated TSH response in the TRH stimulation test, revealing true hypothyroid state and one-third (34%) of SCH patients showed normal response. Mojiminiyi et al [30] performed TRH stimulation test in 34 SCH patients and 12 (35%) patients showed a normal response to the TRH test, compatible with our data.

The proportion of subjects demonstrating an exaggerated TSH response to TRH exhibited significant increasing trend as basal TSH levels increased, compatible with previous report [27].

The role of autoantibody and higher basal TSH level to predict the increased incidence of the future hypothyroidism in SCH was reported in several cohort studies [2, 3, 5, 29]. And the incidence of positive autoantibodies was reported to increase as TSH level increased [4]. In a 20-year follow-up of the Whickham study [2], basal TSH above 2 mIU/L increased the probability of developing hypothyroidism and the presence of anti-thyroid antibodies further increased the probability. Walsh et al [29] reported a 13-year follow-up study in which the optimal cutoff for predicting future hypothyroidism was basal TSH above 2.5 mIU/L and in women with positive TPOAb or TgAb, the development of hypothyroidism at follow-up was 12% when basal TSH was 2.5 mIU/L or less, but 55.2% for basal TSH 2.5 to 4.0 mIU/L and 85.7% for basal TSH above 4.0 mIU/L. Asvold et al [3] reported basal TSH was positively associated with the risk of hypothyroidism at 11-year follow-up, and the incidence of follow-up hypothyroidism in women was 1.1% with basal TSH 0.50 to 1.4 mIU/L, but increased to 31.5% with basal TSH 4.0 to 4.5 mIU/L and also, in men, the incidence increased from 0.3% to 14.7% with increasing basal TSH.

All of these follow-up cohort studies addressed the risk of future hypothyroidism and not the state of present hypothyroidism. Moncayo et al [28] reported that in the evaluation of 2570 TRH stimulation tests in subjects with normal basal TSH level, exaggerated TRH stimulation tests were found in 5.4% for TSH below 2.0 mIU/L, 30.2% for TSH between 2.0 and 3.0 mIU/L, 65.5% for TSH between 3.0 and 3.5 mIU/L, and 87.5% for TSH between 3.5 and 4.0 mU/L, suggesting latent hypothyroidism was present in normal basal TSH levels and proposed gray area values between 3.0 and 3.5 mIU/L. In our study, SCH was defined with basal TSH levels above 4.0 mIU/L, so direct comparison cannot be made, but the finding of increasing trend of positive TRH stimulation test according to the increasing basal TSH level was compatible with our data. In our results, even with basal TSH levels of 4 to 5 mIU/L, exaggerated TRH stimulation test was positive in 59% of the subjects, suggesting some of the subjects with normal TSH level below 4.0 mIU/L might show an exaggerated TRH stimulation test, as apparent in the report from Moncayo et al [28].

Spencer et al [4] reported that TPOAb prevalence was lowest (< 3%) when TSH was between 0.1 and 1.5 mIU/liter in women and between 0.1 and 2.0 mIU/liter in men and progressively increased to above 50% when TSH exceeded 20 mIU/liter. Our study revealed the incidence of positive TPOAb or TgAb tended to increase as basal TSH levels increased (*P* for trend <0.05) (Table 3, Fig. 4) and in Group6, 54.5% of the patients showed positive TPOAb or TgAb while in Group1, 23.5% of the patients showed positive TPOAb or TgAb, compatible with the previous report [4].

Our data showed that the incidence of autoantibodies was higher in the patient group with positive TRH stimulation test (Table 1). Mojiminiyi et al [30] reported that 11 (44%) of 25 patients with positive anti-thyroid antibody tests and 3 (33%) of 9 patients with a negative antibody screen had hypothyroid responses to TRH, compatible with our data.

Most cohort studies predicted the role of TSH and autoantibody in future hypothyroidism, but few previous reports were available regarding the predictive ability of basal TSH and autoantibodies to predict the present hypothyroid state with an exaggerated TRH stimulation test in SCH. In our analysis of predictive ability, performance of basal TSH to predict positive TRH stimulation test was poor with low sensitivity (65.2%) and specificity (48.8%), and these results seemed to reflect only a weak positive correlation between basal TSH and ΔTSH (*r* = 0.320, *P* < .001, Fig. 2). Mojiminiyi et al [30] reported basal TSH alone was poorly correlated with the response to TRH, compatible with our results. Our data revealed that positive TPOAb and TgAb showed high specificity (more than 90%) to predict positive TRH stimulation test but low sensitivity (19.4% & 20.5% respectively). Thus, positive autoantibodies looked to predict high probability of positive TRH stimulation test, suggesting present hypothyroid state.

The treatment of SCH is still debated [20, 21]. One study revealed that among subjects with SCH, 92% of women with basal TSH lower than 10 mIU/L presented positive TPOAb, or at least one classical cardiovascular risk factor and at least one symptom or sign of hypothyroidism, suggesting the need for consideration of treatment [31]. Our data showed that overall, about two-thirds (66%) of SCH patients showed exaggerated positive TRH stimulation test revealing a hypothyroid state, and even among those with basal TSH 4.00 to 5.00 mIU/L, 59% of the subjects showed exaggerated TRH stimulation test. Also, our results showed that overall one-third of SCH patients showed normal results in TRH stimulation test, and this point should be considered when interpreting the results of the clinical study regarding SCH, which usually comprise up to a third of subjects with normal thyroid function. It might render statistically significant results to nonsignificant results and lack of definitive evidence in the study of SCH. The main limitation of our study is its retrospective nature. We also lacked information about symptoms for thyroid dysfunction. In addition, the possibility of selection bias could not be excluded because the TRH stimulation test could not be performed in all subjects with SCH.

In summary, exaggerated TSH responses to the TRH stimulation test were observed in two-thirds of SCH subjects with TSH levels between 4.01 and 10.00 mIU/L. Positive autoantibodies looked to mean high probability of positive TRH stimulation test but basal serum TSH level showed low predictability for positive TRH stimulation test and this was not sufficient for identifying a hypothyroid state in SCH patients. The decision to treat SCH with basal serum TSH less than 10.00 mIU/L seems to be tailored to the individual patient, and the TRH stimulation test may be a helpful guide in making treatment decisions.

## Conclusion

Our study suggested that two-thirds of SCH showed exaggerated TSH responses in the TRH stimulation test, revealing hypothyroid state. Positive TPOAb or TgAb showed high specificity (more than 90%) for positive TRH stimulation test. Basal serum TSH levels showed poor predictability to positive TRH stimulation test. The TRH stimulation test may be a valuable guide in identifying a hypothyroid state in patients with SCH.

## Data Availability

Some or all datasets generated during and/or analyzed during the current study are not publicly available but are available from the corresponding author on reasonable request.

## Abbreviations

fT4: free thyroxine
ROC: receiver operating characteristic
SCH: subclinical hypothyroidism
TgAb: anti-thyroglobulin antibody
TPOAb: anti-thyroid peroxidase antibody
TRH: thyrotropin-releasing hormone
TSH: thyrotropin (thyroid-stimulating hormone)
